# An integrated distribution scheduling and route planning of food cold chain with demand surge

**DOI:** 10.1007/s40747-022-00811-9

**Published:** 2022-07-18

**Authors:** Youhua Chen, Hongjie Lan, Chuan Wang, Xiaoqiong Jia

**Affiliations:** 1 Beijing Jiaotong University, Haidian District, Beijing, 100044 People’s Republic of China; 2 China Mobile Group Beijing Co.,Ltd, Xicheng District, Beijing, 100032 People’s Republic of China

**Keywords:** Intergraded optimization, Order processing, Route planning, Heuristic algorithm

## Abstract

With the rapid development of e-commerce, customers could order online to ensure timeliness. Therefore, e-commerce enterprises need to pick and distribute customers’ orders. These two operations are interdependent. Order picking needs to consider the vehicle route planning. At the same time, the vehicle route planning is also based on the batching of orders. Considering the demand surge scenario of food cold chain, with the shortest time and lowest cost to complete all distribution tasks as the objective, this paper aims at the integrated optimization of distribution scheduling and route planning, and establishes a mixed integer programming mathematical model. Finally, we design a three-stage heuristic algorithm to solve this problem, and use the actual data to carry out numerical experiments to verify the reliability and effectiveness of the mathematical model and heuristic algorithm.

## Introduction

Demand surge refers to the unpredictable increase in the operating system where the demand caused by emergencies exceeds the upper limit of normal demand fluctuations [2]. At present, researches on demand surge mainly include natural disasters [25], public health events [16], and e-commerce promotional shopping [14]. In terms of e-commerce promotion, the concentrated shopping behavior of customers will trigger a demand surge. An e-commerce platform has completed a transaction volume of 498.2 billion yuan during the “Double Eleven” promotion in 2020. According to the group’s financial report, the total transaction volume in 2020 is 3.202 trillion yuan, a year-on-year increase of 23%. The average daily transaction volume is 8.773 billion yuan, and the average daily sales during the “Double Eleven” promotion period in 2020 will be more than 5 times as the annual average daily turnover. According to data from the State Post Bureau, on the day of the “Double Eleven” e-commerce platform promotion in 2020, the nationwide processed postal (express) items reached 675 million, a year-on-year increase of 25.8%. This shows that large-scale e-commerce promotions will cause demand surge.

For e-commerce enterprise, processing a large number of orders in a short period puts pressure on merchant logistics activities [17]. Delays in delivery time, warehouse explosion, and untimely feedback of logistics transportation and logistics information emerge in an endless stream. The proliferation of express parcels has brought enormous pressure to the management of distribution centers [15]. During the COVID-19 period, measures such as home isolation caused residents to purchase materials in large quantities in a short period of time, triggering a large-scale demand surge. At present, most businesses have encountered problems such as accumulation of goods in warehouses, slow picking, and insufficient own transport capacity. In order to meet the surging demand of consumers in the short term, merchants need to respond quickly and accurately, which brings severe challenges to related logistics activities.

Order picking and vehicle delivery are two important parts of logistics activities, and these two parts are interrelated. In order to improve the efficiency of logistics activities and improve customer satisfaction, picking and delivery decisions need to be made simultaneously. Chen [3] pointed out that greater cost savings can be achieved through integrated research instead of improving individuals. Chen and Vairaktarakis [5], Park and Hong [23], Ullrich and Christian [28] also pointed out that integration can lead to an average improvement of 5–20% compared with independent optimization methods.

In the context of consumption growth, the demand for cold chain food continues to rise, especially festivals such as promotions. Both SF Express and JD Fresh said that fresh food is the most popular among all sales categories. Whether relying on third-party logistics or self-built logistics, there will be problems such as delayed delivery and high cargo damage rate when there is demand surge. Cold chain logistics is an important way to ensure food safety and improve product quality, especially in the transportation and delivery stages where products are prone to damage, safety and logistics efficiency are particularly important.

Difficulty, slowness, and high cost have always been the pain points of cold chain logistics. The problem of chain disconnection often occurs. Cold chain food orders have characteristics of delivery destinations scattered, small demand while great types for items within a certain order, and high transportation cost due to temperature control, which will increase the difficulty of distribution scheduling and route optimization, and specific problems such as high cost of fulfillment, long fulfillment time and even overtime appear:

(1) Some orders cannot be processed in time

The rapid growth of demand in a short time may cause “explosion of warehouses” and other phenomenons, e.g. irregular and untimely distribution operation, order arrivals exceeding processing capacity, etc. Order batching and sequencing cannot be dealt timely, this results in the accumulation of orders, which makes some orders unable to proceed normally, which in turn affects the subsequent series of operations.

(2) Long order fulfillment time

Due to the large increase of orders in a short period, it has caused certain delivery difficulties for food cold chain delivery companies. For example, the increase in order demand and the growth rate in the width and depth of SKU lead to slow sales, and further slower delivery during e-commerce promotion. In addition to the difficulties and pains of delivery under regular demand conditions, there are more considerations, companies have limited resources, and the demand surge leads to insufficient capacity, which may require external assistance, such as vehicles and manpower. Under demand surge, companies need to take measures to ensure the delivery time of orders. If external forces are not used and their own capacity is insufficient, the order fulfillment time will become longer, which will increase order time penalty and damage to the company’s own reputation and will further reduce customer satisfaction.

(3) High delivery costs

During demand surge, too many orders may lead to decrease in processing capacity, backlog of orders, and longer order fulfillment time. Due to the special nature of fresh food, the quality of fresh food cannot be guaranteed, and corresponding damage cost will increase; while demand surges create more manpower, materials and resources which caused an increase in labor costs, fixed costs, etc.; meanwhile there is an increase in delivery costs due to unreasonable route planning. It can be seen that the demand surge has caused an increase in delivery cost of all aspects, so that the cost remains high, which is also a point that is more difficult for enterprises to overcome.

**Fig. 1:**
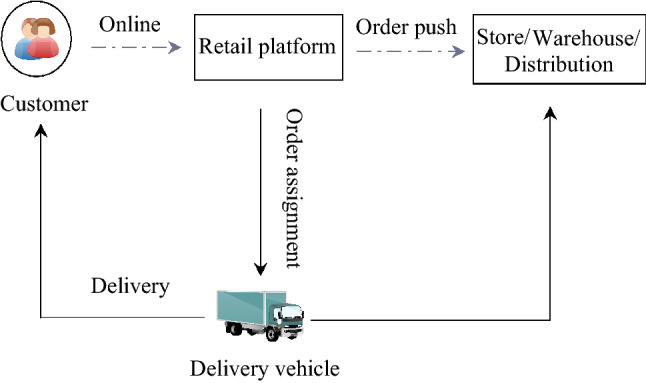
B2C distribution service process

To sum up, whether they can coordinate cold chain food scheduling and delivery through optimization, and quickly pick, package and deliver them to customers with a shorter service time and lower cost is the problem that needs to be solved urgently.

This paper studies the cold chain food distribution problem under demand surge, considering the order batching and sequencing problem in the distribution scheduling link and the route optimization problem in delivery link, and optimizes with the shortest time and the lowest cost. Figure 1 shows the B2C delivery service process. Consumers place orders online, and the retail platform processes and distributes cold chain food orders, which are then delivered to consumers by vehicles. The contribution of paper mainly includes the following two points:

- This paper is the first article that introduces demand surge into the food cold chain distribution problem, and jointly optimizes distribution scheduling and route planning of food cold chain;
- This paper not only proposes a three-phase heuristic algorithm for the research problem, but also analyzes the company’s actual data. The research helps to improve the existing distribution model of cold chain food.

## Literature review

### Demand surge

Many studies have addressed the surge in demand. Olsen and Porter [20] reviewed demand surges and discussed their definitions, models, and common research themes. Huang et al. [9] pointed out that demand surges are significant demand increments in addition to regular demand. The demand surge is mostly caused by emergencies, such as man-made events and natural disasters, such as terrorist attacks, earthquakes, disease outbreaks, volcanoes, floods, storms, etc. These disasters can trigger massive demands for humanitarian products such as medicines, food, water, etc. [26]. In recent years, the problem of demand surge caused by promotional activities has gradually begun to be studied [14].

Due to demand surge, the picking operation of some orders may be delayed or adjusted due to limited resources (such as driver’s working time limit, insufficient self-owned vehicles, etc.) and capacity constraints, while the delayed adjustment will affect the subsequent distribution services and route planning, coupled with the lack of its own capacity, which will result in demand delays. The solution to demand surge is generally to increase picking efficiency or add sufficient distribution capacity. Order picking is one of the important activities of warehousing operations and has an important impact on improving the efficiency of warehouse operations [31]. Liu et al. [14] proposed delivery capability sharing to provide delivery capacity. For the capacity constraint problem, Naber and Kolisch [19], Peteghem and Vanhoucke [24] studied the scheduling problem of multiple delivery execution modes considering capacity constraints. In response to demand surge, there are also other directions of research: supply chain coordination management [13, 27] or inventory control and location problems [1], which will not be discussed in detail here. In this paper, by adjusting the order batching, batching sequence and path planning, the joint decision of delivery scheduling and vehicle routing can be made efficiently and accurately.

### Integrated optimization

At present, there are many related papers on integrated optimization of delivery scheduling and routing [6, 33], and some reviews can be referred to Chen [4], Moons et al. [18]. The integration research of delivery scheduling and routing can be abstracted as the production and delivery scheduling problem, which includes two stages: production scheduling and delivery scheduling: production scheduling solves when each order is processed and the processing time required. In the delivery stage, when each order is delivered, the number of vehicles required for delivery, vehicle routing and delivery time, etc. [29]. There are also most scholars who study the two stages separately. while they often cannot obtain better solutions due to constraints, and so on, integrated research can obtain suboptimal solutions [18]. And solutions that are favorable for order picking are not necessarily equally favorable under integrated optimization decisions. Therefore, the joint optimization of order picking and vehicle routing is very important.

Wei et al. [30] studied production scheduling and distribution planning decisions that considered the two-stage production process at the same time, making the integrated decisions more beneficial than solved in the hierarchical process. For most production enterprises, order scheduling means production scheduling, this paper is oriented to distribution enterprises, and only considers the order process in distribution center, that is order batching, batching sequence and route planning. Jiang et al. [10] studied the order acceptance and scheduling problem whit batch delivery, and developed two approximate algorithms to solve this NP-Hard problem.

However, there are some differences between the problem of integrated delivery scheduling and vehicle routing and the problem of integrated production scheduling and vehicle routing: (1) In the scheduling stage, orders in the e-commerce environment have the characteristics of small batches and high frequency, so it is necessary to use order batching strategy to improve picking efficiency [24]; (2) The problem of integrated production scheduling and vehicle routing is batching production and batching delivery. It is generally assumed that the same vehicle will delivery after the completed batching production. While different order batching may be delivered by the same vehicle in the problem of integrated delivery scheduling and vehicle routing; (3) The problem of integrated production scheduling and vehicle routing generally aim to minimize the order completion time, while the problem of integrated delivery scheduling and vehicle routing generally consider the two objectives of time and cost to be optimized at the same time, which increase the complexity.

In order to meet customer expectations for fast and low-cost delivery, Moons et al. [17] proposed a record-to-record travel algorithm to solve this problem considering both warehouse and distribution operations. The results show that integrated research can improve service levels, both by reducing the time between placing an order and receiving the goods, and by saving average 1.8% in costs. Kuhn et al. [12] comprehensively considered the problem of order batching, order picking, and delivery operations, and proposes a new modeling and solution approach to address practically relevant problem sizes, further proposing the well-known Adaptive Large Neighborhood Search (ALNS) metaheuristic that we call General ALNS (GALNS), showing that the GALNS method outperforms similar ALNS algorithms in 96.35% of the generated problem instances.

### Food cold chain logistics

Considering the requirements of fresh food preservation and temperature control, the distribution of fresh products in this paper is based on the particularity of fresh products, and the distribution is scheduled in units of orders. And the distribution vehicles of food cold chain are generally different from ordinary vehicles. Vehicles may carry different fresh foods for specific product categories, including multiple different spaces for storage of non-mixable fresh products with different storage temperature requirements [22]. Chen [4] conducted research on make-to-order and fresh products, analyzed the current integrated scheduling model of production and outbound distribution, and presented a unified model representation scheme, classified existing models into several different classes for each class of the models give an overview of the optimality properties, computational tractability, and solution algorithms for the various problems studied in the literature.

Based on the characteristics of cold chain food, many scholars analyze it from the perspective of the time window required by customers. Osvald and Stirn [21] proposed a nonlinear mathematical model to consider production scheduling and vehicle routing optimization problems for perishable food products with time window. Devapriy et al. [6] studied the integration problem of perishable food considering the product life, which must be produced and delivered before it can not be used. Some scholars have studied from the perspective of high cost of cold chain logistics, aiming at cost minimization. Yao et al. [32] studied the vehicle routing problem of fresh seafood, minimized the total cost of delivery, and used ant colony optimization to solve the problem. Hu et al. [8] studied the path optimization problem of refrigerated trucks in the distribution process of fresh products, considering the impact of refrigeration cost, operating cost, time loss, and cargo damage, an adaptive heuristic method is proposed, which divides the path and time scheduling into two stages, and commbined variable neighborhood search and particle swarm optimization algorithm are used to solve.

In conclusion, existing studies have studied the integrated problem of cold chain food, but their research is not applicable to the situation of demand surge, especially in the case of demand surge, how to reasonably plan delivery scheduling and vehicle routing optimization to deliver cold chain food in the short term. This paper combines demand surge with integrated study of cold chain foods.

The rest structure of this paper is as follows: The third part describes the problem and determines the optimization objective with the shortest time and the lowest cost for modeling; the fourth part presents the design scheme of three-phase heuristics algorithm; The actual data of the e-commerce platform is solved, and the research results are obtained by data analysis; finally, the conclusions are explained in the sixth part.

**Fig. 2:**
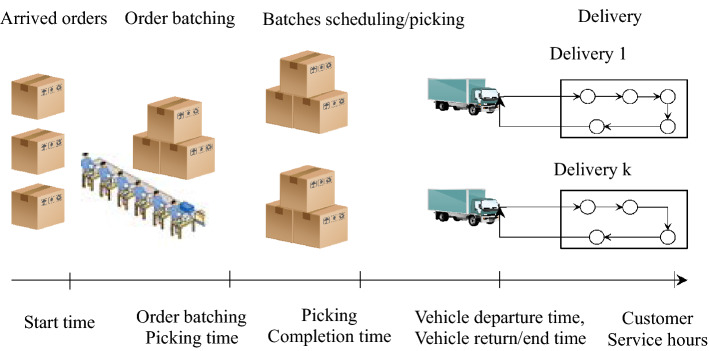
Offline order distribution scheduling and path joint optimization system diagram

## Problem statement and formulation

The order sorting and route optimization described in this article are all based on the company’s self-built logistics distribution model. First, the customer orders need to be sorted in batches and batches according to the order volume and order time window, and then the optimal delivery route is determined according to the customer’s delivery address. Assuming that each customer has only one number of orders per day, and the research object is the number of *n* orders of *n* customers accumulated by the company in a day, construct a model for joint optimization of order batch sorting and distribution routes, and formulate the optimal solution plan, which efficiently completes the delivery of all customer orders. Order demand *n* under surge demand follows a Poisson distribution, $n \in [a, b]$, and a is strictly greater than *M*.

The problem that the model needs to solve is to determine how customer orders are divided into batches, the order of batches, that is, the delivery schedule, and the optimal route for the order delivery vehicle.

When the order arrives, the order is integrated according to a certain batching rule to form multiple batches, and then the batches are sorted according to certain rules, and then different order batches are assigned to the corresponding vehicles for delivery, and the vehicle path optimize. Each vehicle needs to reach multiple customer demand points in a delivery process, so the order and route of arriving customers are planned. The two key factors that are mainly considered in the whole problem are distribution cost and order fulfillment time. Among them, distribution cost includes order distribution scheduling cost, total fixed cost of vehicles, transportation cost, cost of goods loss, time Penalty costs.

In order to meet the above problems, this article makes the following assumptions:

- In the joint optimization problem considered in this chapter, the order information has been obtained in advance;
- The vehicle needs to serve multiple customers every time it departs, so it is necessary to plan the order of customer visits and the distribution path, which consists of a distribution center and n customers, as shown in Fig. 2;
- Assuming that an order can only be delivered by one vehicle, no order splitting is performed;
- Assuming that during the surge demand, all the vehicles used for delivery orders are of the same type, with limited capacity, but the company’s own resources are limited, and measures such as renting vehicles can be taken;
- There are multiple orders for one batch, and multiple batches for one car, but one batch of orders is only served by one car, and one order can only belong to one batch;
- Assuming that each customer has only one order per day.

### Parameter settings

*Settings:*

Set of orders: $N=\{1,2,...,n\}$;

Set of orders with DC: $N_{o}=\{0,1,2,...,n\}$;

Set of vehicles: $V= \{1,2,...,u,u+1,...,w\}$; [1, *u*] are enterprise-owned vehicles, $[u+1,w]$ are leased vehicles.

Set of order batching: $H=\{1,2,...,h\}$;

Set of delivery routing: $R=\{1,2,...,r\}$;

Set of delivery areas: $O=\{1,2,...,o\}$;

*Parameters:*

$q_i$: The quantity of items included in order *i*, $i \in N$;

$Q_h$: Each batch *h* can hold the maximum amount of items, $h \in H$;

$$Q_v $$: Maximum capacity (load capacity) of vehicle *v*, $$v \in V$$;

$t_{ij}$: The time required for the delivery vehicle from delivery point *i* to delivery point *j*, $i,j\in N_0$;

$T_i$: Average service time required for each delivery point *i* (ie each customer);

$t_i^{\mathrm{earliest}}$: The earliest time that delivery point *i* (customer *i*) can accept the order to be delivered;

$t_i^{\mathrm{latest}}$: The latest time that delivery point *i* (customer *i*) can accept the order to be delivered;

$t_i^{\mathrm{arrive}}$: Time when customer order *i* arrives;

$t_i^{\mathrm{arrive'}}$: The actual time when the vehicle arrives at the delivery point *i* (customer *i*);

$d_{ij}$: The distance between delivery point *i* and delivery point *j*;

$l_v^{\max }$: The farthest mileage of vehicle *v*;

$c_i^{\mathrm{pick}}$: Order *i* pick and determine the average cost of the batch; (/unit time);

$c_u^{\mathrm{fixed}}$: Fixed cost of own vehicle *u* per using;

$c_w^{\mathrm{fixed}}$: Fixed cost of renting a vehicle *w* per using;

$c_u^p$: Unit transportation cost of own vehicle *u*;

$c_w^p$: Unit transportation cost of renting vehicle *u*;

$c_i^{\mathrm{earliest}}$: The unit penalty cost for an order earlier than the earliest acceptable time by the customer;

$c_i^{\mathrm{latest}}$: The unit penalty cost for an order later than the latest acceptable time by the customer;

$\alpha$: Proportion of cargo damage during transportation;

$\beta$: Proportion of cargo damage during loading and unloading;

$\theta$: Average cost of goods damage of the order;

*Decision variables:*

$t_h^{\mathrm{start}}$: Start Picking time of batch *h*,$h\in H$;

$t_h^{\mathrm{sevice}}$: All the time required to complete the picking of batch h, $h\in H$;

$t_i^{\mathrm{complete}}$: Picking completion time of order *i* (the batch);

$t_v^{\mathrm{leave}}$: Time when vehicle *v* left the distribution center;

$t_v^{\mathrm{end}}$: The total time for vehicle *v* to complete a delivery service;

$r_i$: Flow of distribution point *i*;



$$y_{hv} = {\left\{ \begin{array}{ll} 1, &{} \text {if order batching }h\text { is allocated to vehicle }\\ &{}\quad v\text {(including own vehicles } u\text { and rented}\\ &{}\quad \text {vehicles }w\text {);} \\ 0, &{} \text {otherwise};\quad {h=1,\dots ,H, v=1,\dots ,w.} \end{array}\right. }$$

$$z_{ijv} = {\left\{ \begin{array}{ll} 1, &{} \text {if vehicle }v\text { is delivered from distribution}\\ &{}\quad \text {point }i\text { to distribution point }j\text {;} \\ 0, &{} \text {otherwise};\quad {i=1,\dots ,n, j=1,}\\ &{}\qquad \qquad \quad {\dots ,n, v=1,\dots ,w.} \end{array}\right. }$$

$$\tau _{io} = {\left\{ \begin{array}{ll} 1, &{} \text {if the service address of order }i\text { belong to}\\ &{}\quad \text {delivery area }o\text {;} \\ 0, &{} \text {otherwise};\quad {i=1,\dots ,n, o=1,\dots ,O.} \end{array}\right. }$$

$$\delta _{hr} = {\left\{ \begin{array}{ll} 1, &{} \text {if order batching }h\text { is allocated to delivery}\\ &{}\quad \text {route }r\text {;} \\ 0, &{} \text {otherwise}; \quad {h=1,\dots ,H, r=1,\dots ,R.} \end{array}\right. }$$

$$\sigma _{or} = {\left\{ \begin{array}{ll} 1, &{} \text {if delivery route }t\text { belong to delivery area }o\text {;} \\ 0, &{} \text {otherwise};\quad {r=1,\dots ,R, o=1,\dots ,O.} \end{array}\right. }$$

### Model construction

(1) The time function part of the objective function is to minimize the order fulfillment time. The total time for a vehicle to complete a delivery service is the sum of the transportation time of the vehicle for a delivery and the service time to the customer.

1 $$\begin{aligned} \min f_{1}= & {} min_{v \in V} \left\{ t_v^{leave}+\sum _{i \in N} z_{oiv}t_{oi}+\sum _{i \in N}\sum _{j \in N} z_{ijv}t_{ij}\right. \nonumber \\&\left. +\sum _{j \in N}z_{jov}t_{jo}+\sum _{i \in N}\sum _{j \in N} z_{ijv}T_{i}\right\} \end{aligned}$$

Equation (1) is to minimize the order fulfillment time, including the leave time and travel time.

(2) The cost function part of the objective function. The cost function includes order picking and sorting costs, total fixed vehicle costs, transportation costs, cargo damage costs, and time penalty costs.

(1) Order delivery schedule cost

The surge demand will lead to increased costs in the process of order batching, picking, and batch sorting. The cost of order batching, picking and sorting includes picking labor costs, management costs, and technical costs. Therefore, the order picking and sorting cost function is:

2 $$\begin{aligned} C_{1} = \sum _{i=1}^N \sum _{h=1}^H t_{i}^{\mathrm{complete}} c_{i}^{\mathrm{pick}} x_{ih} \end{aligned}$$

(2) Vehicle fixed cost

The fixed cost of a vehicle is related to the vehicle itself and other factors, such as the depreciation expense of the vehicle, the use loss of the vehicle, the management cost of the vehicle, and the cost of the driver. In the case of a surge demand, the company’s own resources are limited, and its own capacity is difficult to meet the delivery requirements. Therefore, the total fixed cost of the vehicle is divided into two parts, one is the fixed cost of the own vehicle, and the other is the fixed cost of the leased vehicle. Whether to use the leased vehicle is determined by the 0-1 variable $z_{ijv}$, which is the same as whether to use the own vehicle. Therefore, the fixed cost of the vehicle is:

3 $$\begin{aligned} C_{2} = \sum _{j=1}^N \sum _{v=1}^u c_{u}^{\mathrm{fixed}}z_{ojv}+ \sum _{j=1}^N \sum _{v=u+1}^w c_{w}^{\mathrm{fixed}} z_{ojv} \end{aligned}$$

(3)Delivery cost

Delivery cost is related to factors such as vehicle transportation distance, delivery time, and vehicle path, and generally has a positive correlation with vehicle transportation distance or transportation time. In this article, an expression that is positively correlated with transportation time is adopted. Same as $C_2$, the cost of transportation vehicles is divided into two parts, one is the transportation cost of the company’s own vehicles, and the other is the transportation cost of the rented vehicles:

4 $$\begin{aligned} C_{3} = \sum _{i=0}^{N_0}\sum _{j=1}^N \sum _{v=1}^u c_{u}^{p}t_{ij}z_{ijv}+ \sum _{i=0}^{N_0} \sum _{j=1}^N \sum _{v=u+1}^w c_{w}^{p} t_{ij}z_{ijv} \end{aligned}$$

(4) Cargo damage cost

Due to the particularity of fresh food, the freshness of fresh food should also be considered as a factor in the model when the cold chain transportation guarantee mechanism in my country is not perfect and the damage is high. As time, temperature, humidity and other external factors change, the quality of food will also change, and the freshness of fresh food will also change due to the difference in warehouse, vehicle, and external environment conditions during loading and unloading. Therefore, the loss cost of fresh food is:

5 $$\begin{aligned} C_{4} = \sum _{i=0}^N\sum _{j=1}^N \sum _{v=1}^V z_{ijv}\theta (\alpha t_{ij}+\beta )q_{i} \end{aligned}$$

(5) Time penalty cost

In the process of order receiving, sorting, sorting, and delivery, it is inevitable that there will be delays in delivery or early arrival. The time penalty cost is as follows:

6 $$\begin{aligned} C_{5}= & {} \sum _{i=0}^N\sum _{j=1}^N \sum _{v=1}^V c_{i}^{\mathrm{earliest}}z_{ijv} \max (t_{i}^{\mathrm{earliest}}-t_{i}^{\mathrm{arrive}},o) \nonumber \\&\quad + \sum _{i=0}^N\sum _{j=1}^N \sum _{v=1}^V c_{i}^{\mathrm{latest}}z_{ijv} \max (t_{i}^{\mathrm{latest}}-t_{i}^{\mathrm{arrive}},o) \end{aligned}$$

In summary, the distribution schedule and path cost part of the model objective function is as follows:

7 $$\begin{aligned} \min f_{2}&=C_{1}+C_{2}+C_{3}+C_{4}+C_{5}\nonumber \\&= \sum _{i=1}^N \sum _{h=1}^H t_{i}^{\mathrm{complete}} c_{i}^{\mathrm{pick}} x_{ih}\nonumber \\&\quad + \sum _{j=1}^N \sum _{v=1}^u c_{u}^{\mathrm{fixed}}z_{ojv}+ \sum _{j=1}^N \sum _{v=u+1}^w c_{w}^{\mathrm{fixed}} z_{ojv}\nonumber \\&\quad + \sum _{i=0}^N\sum _{j=1}^N \sum _{v=1}^u c_{u}^{p}t_{ij}z_{ijv}+ \sum _{i=0}^N\sum _{j=1}^N \sum _{v=u+1}^w c_{w}^{p} t_{ij}z_{ijv}\nonumber \\&\quad + \sum _{i=0}^N\sum _{j=1}^N \sum _{v=1}^V z_{ijv}\theta (\alpha t_{ij}+\beta )q_{i}\nonumber \\&\quad + \sum _{i=0}^N\sum _{j=1}^N \sum _{v=1}^V c_{i}^{\mathrm{earliest}}z_{ijv} \max \left( t_{i}^{\mathrm{earliest}}-t_{i}^{\mathrm{arrive}},o\right) \nonumber \\&\quad + \sum _{i=0}^N\sum _{j=1}^N \sum _{v=1}^V c_{i}^{\mathrm{latest}}z_{ijv} \max \left( t_{i}^{\mathrm{latest}}-t_{i}^{\mathrm{arrive}},o\right) \end{aligned}$$

Since the function $f_1$ and the function $f_2$ have different dimensions, $f_1$ is a time function, and $f_2$ is a cost function. In order to improve the solution efficiency of the model, the model is normalized.

8 $$\begin{aligned} \min F&= \min \omega f_1+ \min (1-\omega ) f_{2}\nonumber \\&= \min \omega \left( \sum _{i \in N} z_{oiv}t_{oi}+\sum _{i \in N}\sum _{j \in N} z_{ijv}t_{ij}\right. \nonumber \\&\quad \left. +\sum _{j \in N}z_{jov}t_{jo}+\sum _{i \in N}\sum _{j \in N} z_{ijv}T_{i} +t_v^\mathrm{{leave}} \right) \nonumber \\&\quad + \min (1-\omega ) \left( \sum _{i=1}^N \sum _{h=1}^H t_{i}^{\mathrm{complete}} c_{i}^{\mathrm{pick}} x_{ih}\right. \nonumber \\&\quad + \sum _{j=1}^N \sum _{v=1}^u c_{u}^{\mathrm{fixed}}z_{ojv}+ \sum _{j=1}^N \sum _{v=u+1}^w c_{w}^{\mathrm{fixed}} z_{ojv}\nonumber \\&\quad + \sum _{i=0}^N\sum _{j=1}^N \sum _{v=1}^u c_{u}^{p}t_{ij}z_{ijv}+ \sum _{i=0}^N\sum _{j=1}^N \sum _{v=u+1}^w c_{w}^{p} t_{ij}z_{ijv}\nonumber \\&\quad + \sum _{i=0}^N\sum _{j=1}^N \sum _{v=1}^V z_{ijv}\theta (\alpha t_{ij}+\beta )q_{i}\nonumber \\&\quad + \sum _{i=0}^N\sum _{j=1}^N \sum _{v=1}^V c_{i}^{\mathrm{earliest}}z_{ijv} \max \left( t_{i}^{\mathrm{earliest}}-t_{i}^{\mathrm{arrive}},o\right) \nonumber \\&\left. + \sum _{i=0}^N\sum _{j=1}^N \sum _{v=1}^V c_{i}^{\mathrm{latest}}z_{ijv} \max \left( t_{i}^{\mathrm{latest}}-t_{i}^{\mathrm{arrive}},o\right) \right) \end{aligned}$$

9 $$\begin{aligned} \text {s.t.:}&\sum _{h \in H}x_{ih}=1, \quad \forall i\in N \end{aligned}$$

10 $$\begin{aligned}&\sum _{i \in N}x_{ih} q_i \le Q_h, \quad \forall h\in H \end{aligned}$$

11 $$\begin{aligned}&t_h^{\mathrm{pick}}=t_h^{\mathrm{start}}+t_h^{\mathrm{service}} \end{aligned}$$

12 $$\begin{aligned}&t_i^{\mathrm{complete}}=\sum _{h\in H}x_{ih}t_i^{\mathrm{pick}},\quad \forall i \in N \end{aligned}$$

13 $$\begin{aligned}&t_v^{\mathrm{leave}}= \max {t_i^{\mathrm{complete}}y_{iv}}, \quad \forall v\in V \end{aligned}$$

14 $$\begin{aligned}&\sum _{i \in N}\sum _{j \in N}q_i z_{ijv}\le Q_v, \quad \forall v\in V \end{aligned}$$

15 $$\begin{aligned}&\sum _{v\in V}y_{iv}=\left\{ \begin{array}{ll} V, &{}i=0\\ 1, &{}i \in N \end{array} \right. \end{aligned}$$

16 $$\begin{aligned}&\sum _{i \in N}\sum _{v\in V}z_{ijv}= \left\{ \begin{array}{ll} V, &{}j=0\\ 1, &{}j \in N \end{array} \right. \end{aligned}$$

17 $$\begin{aligned}&\sum _{j \in N_{0}}z_{ijv} = y_{iv}, \forall i \in N_0.v\in V \end{aligned}$$

18 $$\begin{aligned}&\sum _{j \in N}z_{0jv} = 1.\quad \forall v \in V \end{aligned}$$

19 $$\begin{aligned}&r_0 = 0 \end{aligned}$$

20 $$\begin{aligned}&r_j-r_i \ge (q_i+Q_v)\sum _{v\in V}z_{ijv}-Q_v,\ \forall i \in N_0, j\in N \end{aligned}$$

21 $$\begin{aligned}&r_j \le \sum _{i \in N}\sum _{v\in V}z_{ijv}Q_V,\quad \forall j \in N \end{aligned}$$

22 $$\begin{aligned}&\sum _{i=0}^N\sum _{j=1}^N z_{ijv}l_{ij}\le l_v^{max}, \quad \forall i,j \in N,v\in V \end{aligned}$$

23 $$\begin{aligned}&t_{ij}\ge 0, i,j \in N \end{aligned}$$

24 $$\begin{aligned}&c_w^{\mathrm{fixed}} \ge c_u^{\mathrm{fixed}} \end{aligned}$$

25 $$\begin{aligned}&c_w^p \ge c_u^p \end{aligned}$$

Constraint (9)and (10) are order batch constraints, to ensure that each order can only be allocated to one order batch, and the maximum amount of goods that can be accommodated in each batch does not exceed $Q_b$; Constraint (11) represents the entire time for order batch *h* to complete the picking, including the start picking time and service time; Constraint (12) represents the entire time for the order to be picked; Constraint (13) is the time that the vehicle leaves the distribution center after the batch picking of all orders in vehicle *v* is completed; Constraint (14) represents that the vehicle cannot exceed its maximum vehicle capacity in one delivery; Constraints (15)-(18) are the constraints of the delivery stage. (15) represents that a delivery point *i* is served by only one vehicle *v*; Constraint (16) represents that the vehicle departs from delivery point 0 and finally returns to delivery point 0; Constraint (17) represents that if the vehicle entering the vehicle serves delivery point *i*, it must leave from delivery point *i*; Constraint (18) represents that each car leaves once from the distribution point at 0:00; Constraint (19)-Constraint (21) represents the flow restriction of the distribution point; Constraint (22) represents that the total mileage cannot exceed the maximum mileage of the vehicle; Constraint (23) represents that the delivery travel time of the vehicle is not less than zero; Constraints (24) and (25) are measures for insufficient enterprise’s own capacity under the surge demand; Constraint (24) represents that the fixed cost of a single use of rented vehicles *w* is greater than the fixed cost of a single use of own vehicles *u*, and Constraint (25) represents that the transportation cost of rented vehicle *w* per unit time is greater than the transportation cost of own vehicle *u* per unit time.

## Algorithm

In this section, a three-stage heuristic algorithm is designed to solve the mentioned problem. The first stage uses genetic algorithm to optimize the distribution path, and the second stage gives order batching using the distribution path results, and then adjust the sequence of order batches. The first is to plan the distribution area, use the k-means++ clustering to classify customers, and use the genetic algorithm to optimize the path for each region; then, according to the time window and order batch capacity, order batching is optimized; the last step is to sequence the order batches. Among them, the route planning optimization method determines the vehicle delivery time $t_v^{\mathrm{travel}}$, the order batching optimization determines the time $t_h^{\mathrm{sevice}}$, and the start picking time of different batches determines $t_h^{\mathrm{start}}$. Specific steps are as follows:

**STEP 1: Optimization of route planning**

(1) Customers clustering

Use K-means++ clustering algorithm to partition customers Divide customers into *K* regions, $N = \{r_1,r_2,...,r_k\}$. The objective function is as follows:

26 $$\begin{aligned} \min \sum _{k=1}^K \sum _j d_{koj}^s \end{aligned}$$

Here, according to the research by Ha and Moon [7], Korayem et al. [11], Hu et al. [8] and the survey with enterprise, we use distance as the basis for clustering. The spatial distance between customer *j* and the cluster center can be represented by Euclidean distance, and the distance between actual orders is coordinated. The coordinates of cluster center *k* and customer *j* are $(x_k, y_k), (x_j, y_j )$, and the spatial distance between the cluster center and customer *j* is shown in the following formula (27):

27 $$\begin{aligned} d_{kj}^s=\sqrt{(x_k-x_j)^2+(y_k-y_j)^2} \end{aligned}$$

(2) Route planning

According to the clustering results, we give the optimization of route planning in each region *N*. Genetic algorithm is used to optimize the distribution path to achieve the goal of minimizing the order fulfillment time and cost.

The objective function is stated as follows:

28 $$\begin{aligned} \min F=\sum _v f_v \end{aligned}$$

According to the optimization of genetic algorithm, the travel time of the $v-th$ vehicle can be obtained as $t_v^{\mathrm{travel}}$, and at the same time according to the vehicle speed $V_v$, customer distance and the delivery cost of the $v-th$ vehicle. The function can get the following:

29 $$\begin{aligned} f_v&=\min \omega (C_1+C_2+C_3+C_4+C_5)+(1-\omega )t_v^{\mathrm{travel}}\nonumber \\&\quad +P_1 sign 1+ P_2 sign 2 \end{aligned}$$

In formula (28), $$C_1+C_2+C_3+C_4+C_5$$ is the corresponding cost function, $$t_v^{\mathrm{travel}}$$ is the travel time of the $$v-th $$ vehicle, and *sign*1 represents the current plan. Whether it exceeds the maximum vehicle capacity, and if it exceeds, it is 1, and if it does not exceed it, it is 0. $$P_1$$ is the corresponding penalty coefficient, and generally the value should be greater than the cost function value. *sign*2 indicates whether the current plan meets the customer’s time window requirements, with 0 for satisfaction and 1 for dissatisfaction. $$P_2$$ is the corresponding penalty coefficient, and generally the value should be greater than the cost function value.

**Fig. 3:**
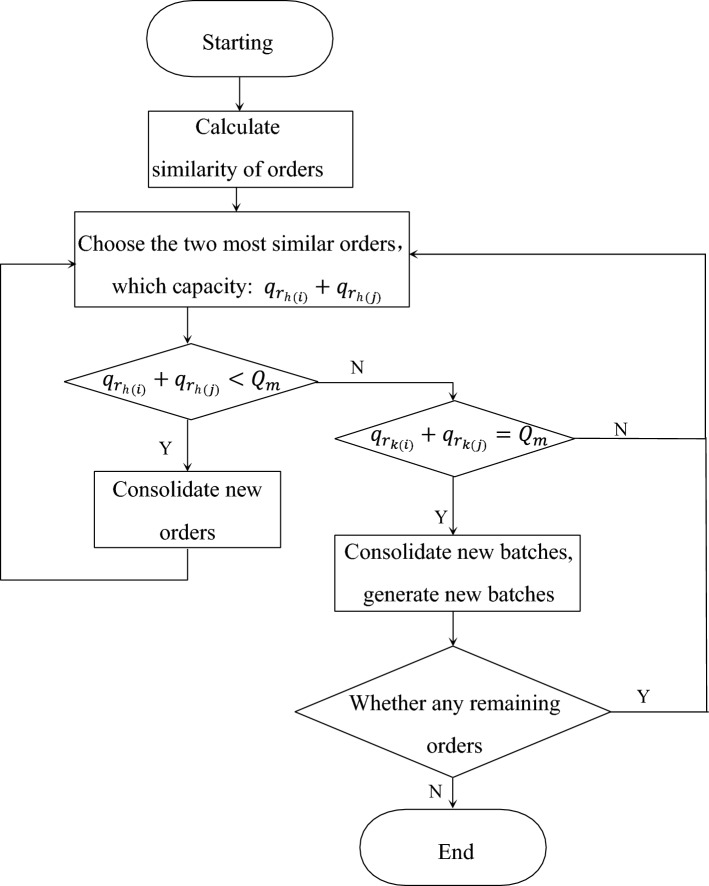
Flow chart of order batching algorithm

The route is optimized by genetic algorithm. Proceed as follows:

(1) Initialize the $t\leftarrow 0$ evolution algebra counter; *T* is the maximum evolution algebra; for all order batches, randomly generate the picking order, and generate a total of *N* individuals as the initial population *P*(*t*);

(2) For individual evaluation, calculate the fitness $f(P(t_i))$ of each individual in *P*(*t*), and the fitness function is 1/*F*;

(3) Selection operation: the selection operator is applied to the group

Selection strategy: Use the selection strategy that combines the best individual preservation and the selection of the gambling wheel. The number of groups *N* in each generation is arranged in ascending order of fitness, and the best-performing individual in the first place is selected, and the best individual is saved according to the selection strategy, and it is copied directly into the next generation, and ranked first in the next generation. Among the $N-1$ individuals of the next generation, based on the fitness of the previous generation group, they are generated by the wheel selection method;

(4): Crossover operation: apply the crossover operator to the group

Using the OX-like crossover method, the crossover operation occurs with a certain probability.

(5): Mutation operation: apply the mutation operator to the group

The mutation operation also occurs with a certain probability. When the mutation operation is generated, the random method is used to generate the number of exchanges *J*, the gene of the individual that needs to be mutated is exchanged *J* times, the position of the exchange gene is randomly generated, and the next generation population is obtained through 3, 4, and 5 operations $P(t +1)$;

(6): Judgment of termination condition $t\le T: t\leftarrow t+1$, go to step 2); $t\ge T$: terminate output solution.

**STEP 2: Optimization of order batching**

The order batching rule based on similarity is used to optimize the order set of the same diatribution route, and the batching rule process is shown in Fig. 3.

(1) Calculate the similarity between orders, the measure of similarity is the order delivery time window interval, and set the maximum interval not to exceed 10 mins;

(2) Select the two orders $r_{h(i)}$, $r_{h(j)}$ with the highest similarity (that is, the closest order time window) in the order set, and the capacity of the two orders is $q_{r_{h( i)}}+q_{r_{h(j)}}$;

(3) Determine whether to merge the batches after constrained judgment:

Judge whether $q_{r_{h(i)}}+q_{r_{h(j)}}<Q_m$ is established: if yes, merge the two orders to generate a new order $r_h$, and return to step (2); if If it is not established, judge whether $q_{r_{k(i)}}+q_{r_{k(j)}}=Q_m$ is established: if yes, merge the two orders to generate a new batch $\varphi _{h(s)}$, then return to step (1); if not, return directly to step 1).

The order set of the distribution route $r_h$ of the $h_{th}$ batch is $N(r_h)$. After step (2) is optimized, the picking batch ${\varphi _h}$ is generated, and the service time is $t_{r_h}^{service}$.

**STEP 3: Sequence adjustment of order batching**

After getting the optimal distribution route and order batching, batching sequence needs to be adjusted to minimize order fulfillment time.

According to the corresponding delivery time $t_h^{\mathrm{travel}}$ in the order batching, the order batches are sequenced with descend order, and the picking sequence of the order baches is obtained.

## Numerical experiments

The data of the case analysis in this article come from the actual operating data of X company. X company was established in 2014. It is a fresh O2O e-commerce platform for the general public and around the general public. Food cold chain distribution company is based on warehouse model. As of June 2018, the scale and number of users of this e-commerce platform have exceeded $50\%$ in the industry, and it is in a leading position in the industry.

Make use of our own advantages to do direct sales and deliver food to customers quickly and well. According to statistical calculations, the e-commerce platform has a total of about 1,200 SKUs, and plans to reach 2,000 SKUs in the future, guaranteeing the quantity and the quality of its fresh food. The e-commerce platform has launched a three-in-one plan of smart logistics, smart supply chain and smart marketing to accelerate the digitalization and intelligence of the entire value chain of fresh food retail, and provide consumers with a better fresh food purchasing and eating experience.

The e-commerce platform actively participates in activities such as centralized discounts and promotions. According to statistics, sales during the Double Eleven in 2019 have increased by 1.7 times year-on-year. Its sales are currently mainly concentrated in first-tier cities, but active users in new first-tier cities are also growing rapidly. As people’s living standards is becoming higher, the market for fresh food and fresh food e-commerce will further expand. Company X began to upgrade its front warehouses in 2019, and developed the company and improved its customer satisfaction by expanding product categories and improving distribution routes with intelligent systems. In the case of a sharp increase in demand and discounts and promotions, the upgrading of the front warehouse has not been completed, and some front warehouses are temporarily unable to meet the delivery needs of all customers. Therefore, some orders are delivered directly from the distribution center. The distribution mode is that the front warehouse and the distribution center are at the same time Distribution.

### Data description and processing

**Fig. 4:**
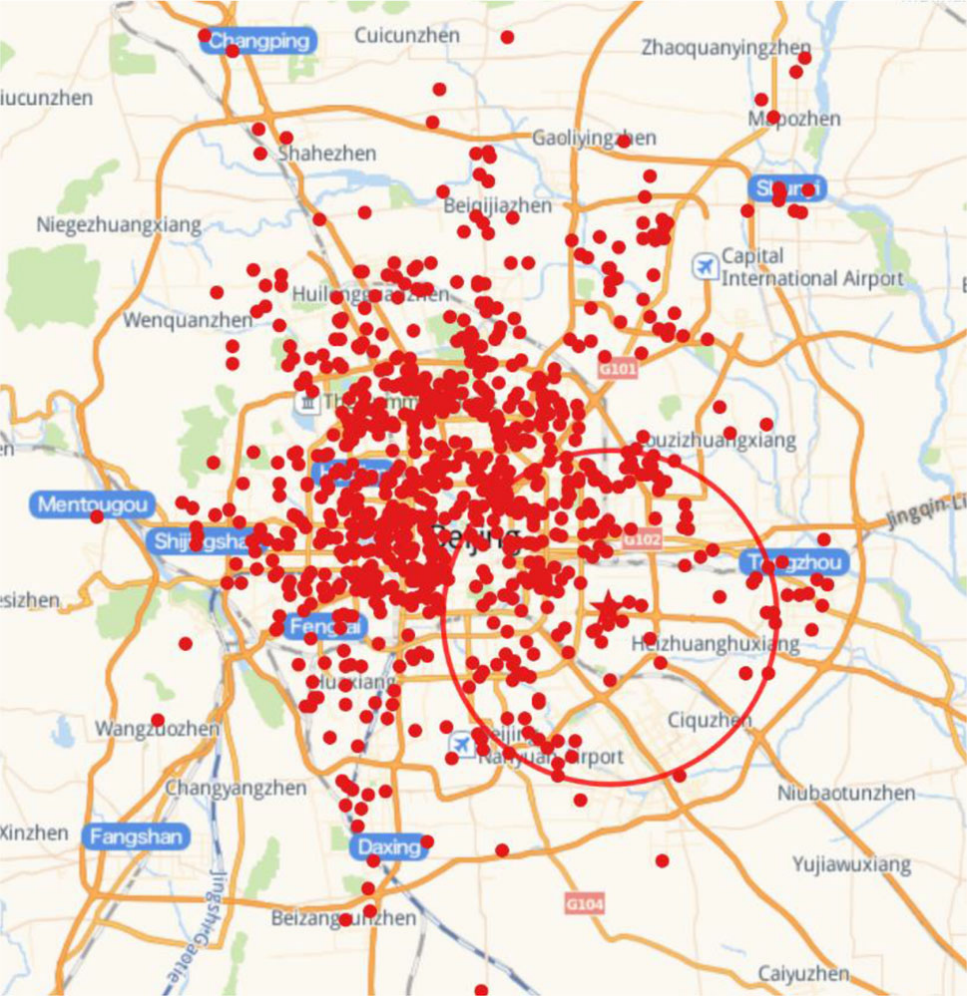
Customer geographic distribution map

(1) Data description This article selects some customer orders from 8:00 a.m. to 14:00 p.m. on a certain day during the promotion period. In the case of a surge demand on this e-commerce platform, some orders are directly delivered from the distribution center to the customer’s point using a delivery vehicle. This paper studies the distribution situation of a distribution center, so the research scope is the customer area covered by a certain distribution center, and the company’s existing terminal distribution vehicle is used for distribution.

(2) Data processing

(1) Location information of distribution centers and customers This article obtains the geographic location information of its distribution centers and customers from X enterprise, a total of 870 customer information. The research scope of this paper is the distribution of customers within 15km of the distribution center, with a total of 203 customers. As shown by the circle in Fig. 4.

Table 1 lists some customer location information.

**Table 1:** Customer’s longitude position

Symbol	Longitude	Latitude	Symbol	Longitude	Latitude	Symbol	Longitude	Latitude
1	116.6264	39.8415	69	116.4266	39.9334	137	116.5042	39.9637
2	116.6264	39.8415	70	116.4663	39.9211	138	116.3892	39.9035
3	116.4632	39.8228	71	116.4606	39.9183	139	116.4464	39.8407
4	116.4626	39.8244	72	116.4537	39.8594	140	116.5105	39.9183
5	116.4085	39.8324	73	116.4397	39.7928	141	116.4946	39.9585
...	...	...	...	...	..	...	...	...
64	116.3893	39.9104	132	116.4380	39.9479	200	116.4778	39.8902
65	116.5264	39.9537	133	116.4660	39.9373	201	116.3847	39.8797
66	116.4355	39.8884	134	116.4766	39.9250	202	116.3834	39.8858
67	116.4193	39.9195	135	116.4188	39.8822	203	116.4603	39.8975
68	116.4390	39.9168	136	116.5020	39.9434	–	–	–

(2) The demand and delivery time window of each customer point

The demand and delivery time window of some customers are shown in Table 2.

**Table 2:** Customer’s demand and distribution time windows

Symbol	Demand	Earliest time	Latest time	Symbol	Demand	Earliest time	Latest time	Symbol	Demand	Earliest time	Latest time
1	7.08	12:00	13:00	69	13.06	12:00	13:00	137	13.27	13:00	14:00
2	17.91	10:00	11:00	70	18.08	10:00	11:00	138	16.39	8:00	9:00
3	10.55	12:00	13:00	71	14.52	8:00	9:00	139	12.54	11:00	12:00
4	14.11	08:00	9:00	72	7.75	12:00	13:00	140	14.92	8:00	9:00
5	19.21	13:00	14:00	73	7.92	8:00	9:00	141	9.43	13:00	14:00
...	...	...	...	...	...	...	...	...	...	...	...
64	17.18	8:00	9:00	132	19.33	12:00	13:00	200	14.83	11:00	12:00
65	7.45	11:00	12:00	133	19.21	12:00	13:00	201	15.41	10:00	11:00
66	19.62	10:00	11:00	134	8.42	13:00	14:00	202	7.29	10:00	11:00
67	12.25	13:00	14:00	135	7.8	8:00	9:00	203	10.96	8:00	9:00
68	10.06	13:00	14:00	136	13.06	13:00	14:00	–	–	–	–

(3) Other parameter settings

In addition to the customer information in Table 1, other parameters are obtained according to the enterprise survey, as shown in Table 3.

**Table 3:** Parameter settings

Parameter	Symbol	Value	Unit
Set of orders	*N*	203	Piece
Set of vehicles	*V*	10(own),unlimited(rented)	Vehicle
Each batch can hold the maximum amount of items	$Q_h$	100	Kilogram
Maximum vehicle capacity (load capacity)	$$Q_v $$	200	Kilogram
Average service time required for each delivery point i (ie each customer)	$T_i$	10	Minute
The farthest mileage of vehicle *v*	$l_v^{max}$	60	Kilometer
Vehicle speed	$V_v$	25	Kilometer/hour
Order i pick and determine the average cost of the batch (/unit time)	$c_i^{pick}$	5	yuan/order
Fixed cost of own vehicle u per using	$c_u^{fixed}$	30	yuan/time
Fixed cost of renting a vehicle *w* per using	$c_w^{fixed}$	50	yuan/time
Unit distribution cost of own vehicle*u*	$c_u^p$	6	yuan/hour
Unit distribution cost of renting vehicle *u*	$c_w^p$	8	yuan/hour
The unit penalty cost for an order earlier than the earliest acceptable time by the customer	$c_i^{earliest}$	5	yuan/hour
The unit penalty cost for an order later than the latest acceptable time by the customer	$c_i^{latest}$	10	yuan/hour
Proportion of cargo damage during transportation	$\alpha$	0.004	–
Proportion of cargo damage during loading and unloading	$\beta$	0.002	–
Average cost of goods damage of the order	$\theta$	20	yuan/kilogram

### Result analysis

(1) Clustering results

**Fig. 5:**
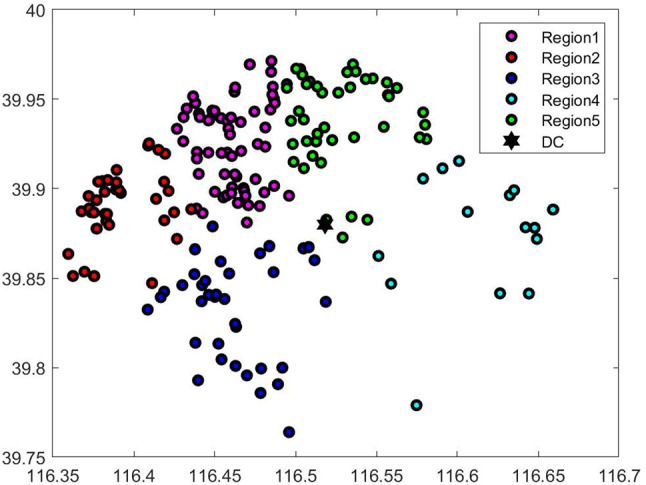
Clustering region division

This article uses K-means++ algorithm for clustering and code the whole program by MATLAB2019b to get the results. Usually, K is set in advance based on people’s experience, and through survey with Company X, we learned that 40-50 customer points are realistic for each distribution area. So, we set the number of clusters to 5. As shown in Fig. 5 below, customer data in this article are divided into 5 areas, which are distinguished by different colors.

**Fig. 6:**
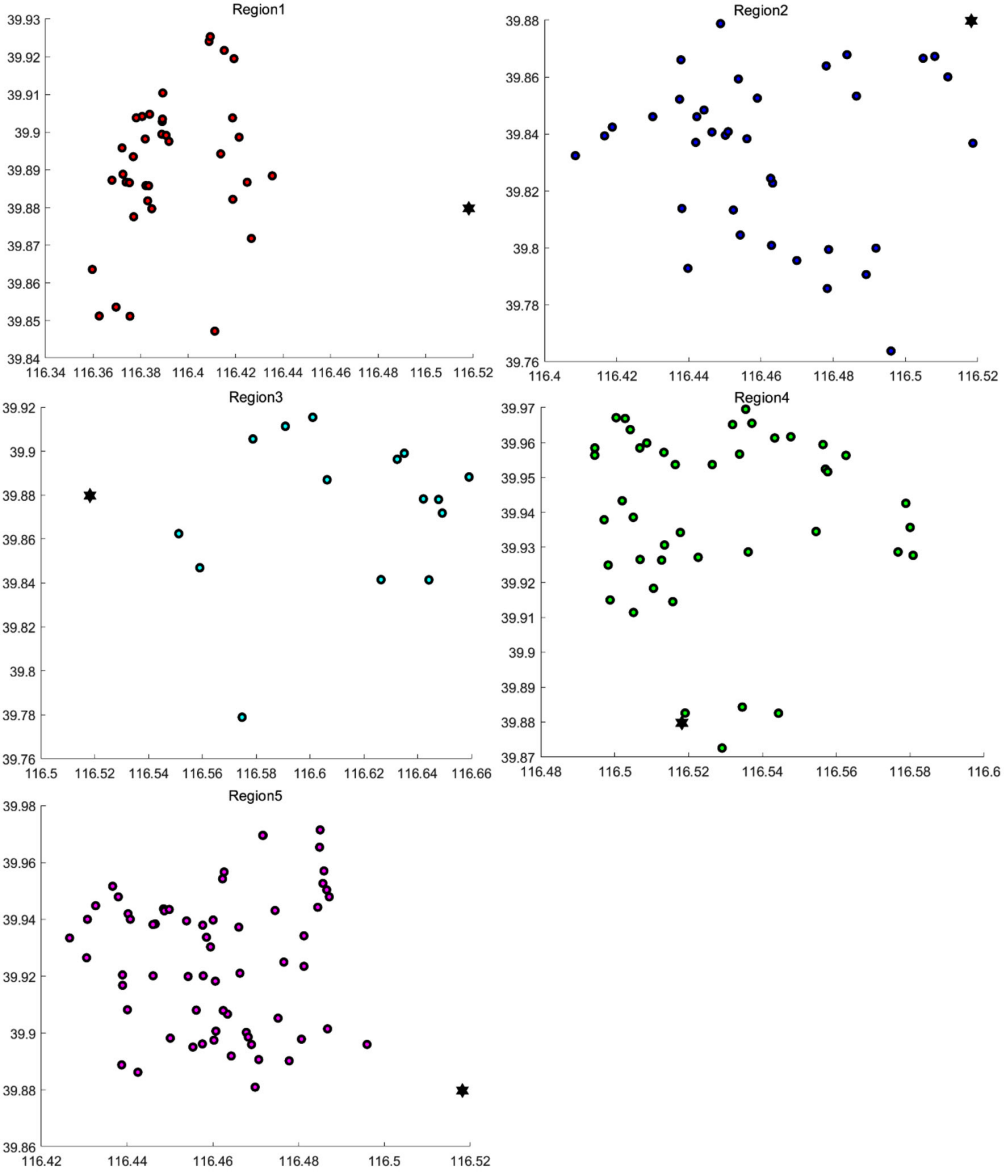
Clustering results

Figure 6 shows the clustering results in each area. Subsequent customer order batching and route planning are carried out in the divided areas.

(2) Delivery route

**Fig. 7:**
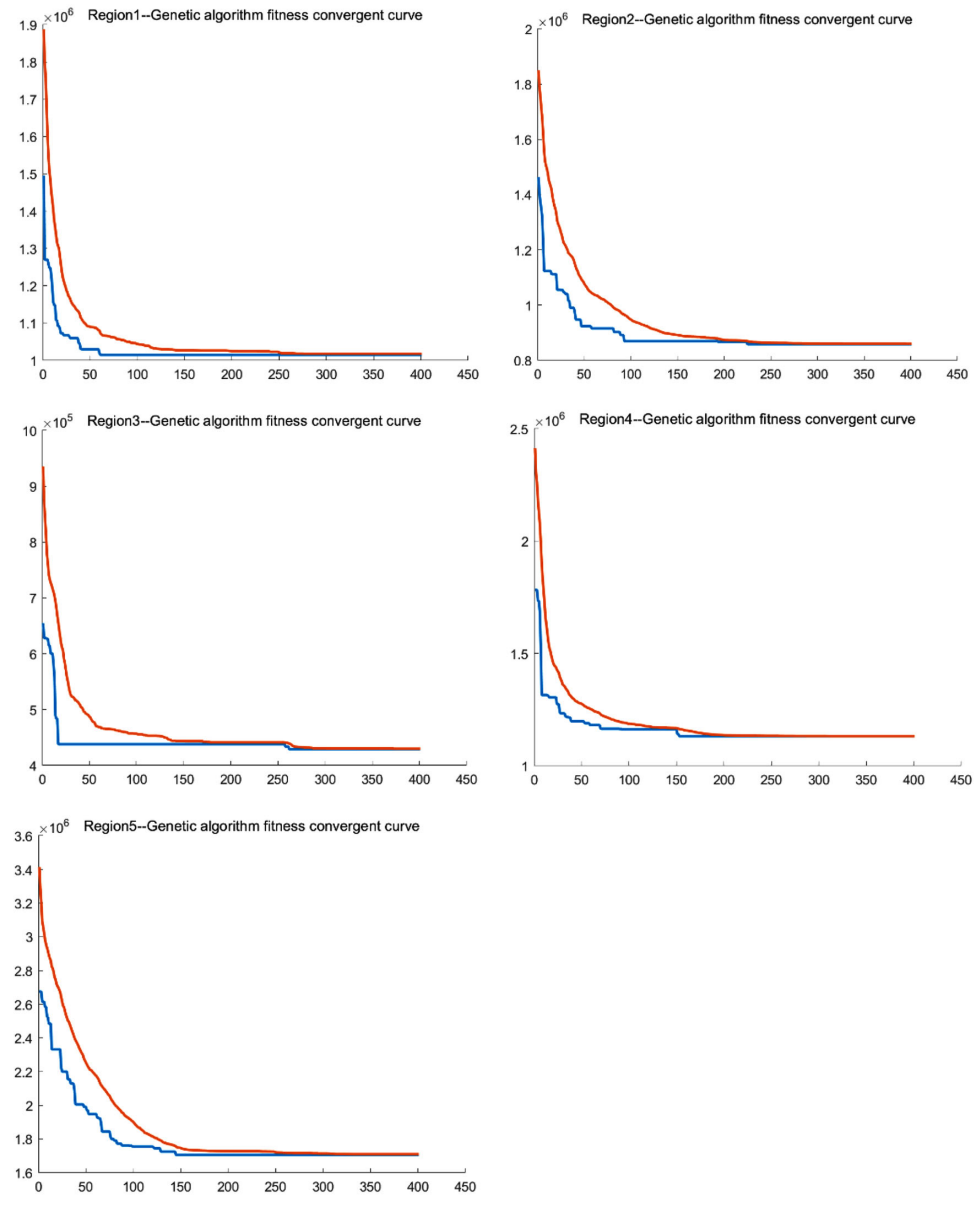
Convergent curve for each region

According to the results displayed by running program, Fig. 7 is the convergence curve of each area, where the initial population is 2000 and the number of iterations is 400, it can be seen that the convergence is basically in progress.

**Fig. 8:**
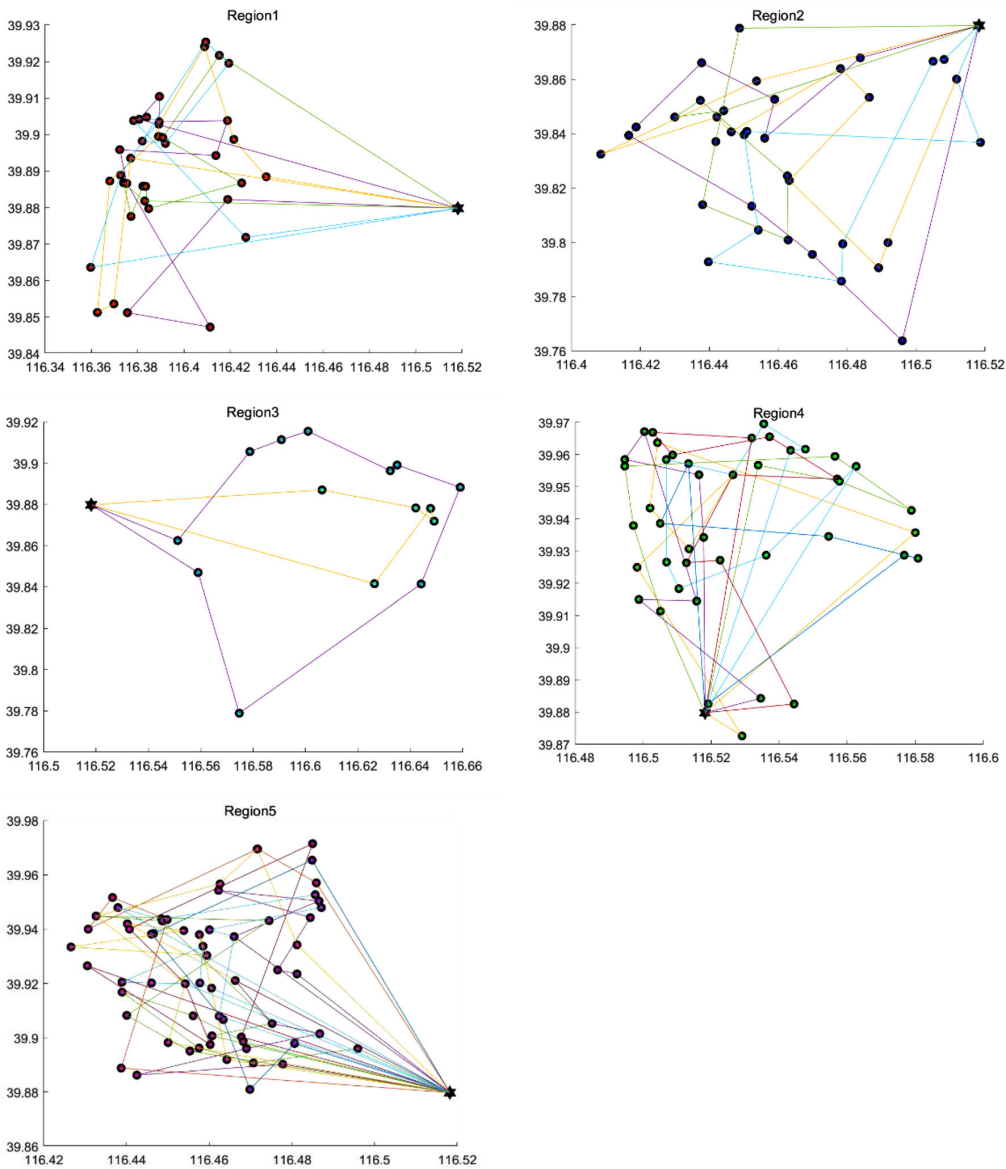
Distribution path for each region

Figure 8 shows the obtained distribution route diagram in each region. There are 38 routes in 5 areas, 4, 4, 2, 6, and 11 routes, respectively. Each color represents a route, which not only shows the customer points of this route, but also gives the result of the delivery sequence.

As shown in Table 4, there are 38 customer points and 4 delivery routes, and each route delivers 9–10 points. The average delivery time is 3.24 hours, the average cost is 136.93 yuan, the average mileage is 39.75 kilometers, and the on-time delivery rate is 89.34%.

Table 5 shows the total time, cost, mileage and load capacity of the route in each region and the sum of all orders. There are 40.6 customers on average in each region. Its delivery time is 5.52–22.69 hours, and the service time is half an hour for each customer. The on-time delivery rate is 87.67%. Moreover, 80% of the vehicle distribution routes have a total load capacity of 50% of the vehicle load.

Due to the cluster analysis based on distance, Region 5 is responsible for the most customers, with a total of 68 customer points. There are 11 delivery routes. However, its average delivery time and delivery cost were 2.06 and 102.33 for each path, less than the average of region 1, which is 3.24 and 136.93.

**Comparison with traditional algorithm**

We address first the order batching, batching sequence, and then route planning as traditional algorithm. The scheduling phase and the routing phase of the traditional algorithm are solved. The total distribution time, total distribution cost, total mileage and vehicle waiting time are obtained. Compared with the three-stage heuristic algorithm in this paper, the results are shown in Table 6.

We analyzed and compared the delivery time and delivery cost of the two algorithms. The three-stage heuristic algorithm showed better performance, with the average delivery time reduced by 5.5% and the average delivery cost by 19%. The main reason is order batching and route planning, in which the total delivery mileage decreased by 5.8%.

**Table 4:** Result analysis of region 1

Route	Total time(hour)	Total cost(yuan)	Total mileage(km)	Total load(kg)
$\left[ 148,152,179,32,50,61,22,145,66 \right]$	3.05	125.87	38.68	159.06
$\left[ 16,64,138,121,122,102,156,181,62,135\right]$	3.43	134.99	44.14	117.48
$\left[ 95,52,195,201,202,182,37,49,114\right]$	2.91	118.50	33.86	115.48
$\left[ 23,31,63,192,153,51,67,103,96,120\right]$	3.56	168.36	42.31	159.71
Total	12.95	547.72	158.99	551.73

From the point of view of total load, the total load has not decreased although the total load of area 2 and area 3 is smaller than that using traditional algorithm. In addition, about the waiting time, the longest vehicle waiting time of the three-stage algorithm is 1.3 hours, while the time of the traditional algorithm is 2.4 hours. Therefore, the three-stage algorithm can effectively improve the picking efficiency and delivery speed, and improve customer satisfaction.

**Table 5:** Total time, cost, mileage and load in each region

Region	Customer number	Total time(hour)	Total cost(yuan)	Total mileage(km)	Total load(kg)
1	38	12.95	547.72	158.99	551.73
2	36	11.59	134.44	125.31	452.11
3	16	5.52	280.20	70.43	209.70
4	45	14.71	892.03	186.10	547.23
5	68	22.69	1125.64	256.99	949.48
Average	40.6	13.49	596.01	159.56	542.05

**Table 6:** Comparion with traditional algorithm

–	Three-phase heuristic algorithm				Tarditional algorithm			
Region	Total time (h)	Total cost (yuan)	Total mileage (km)	Total load (kg)	Total time (h)	Total cost (yuan)	Total mileage (km)	Total load (kg)
1	12.95	547.72	158.99	551.73	14.15	623.92	183.62	545.73
2	11.59	134.44	125.31	452.11	12.36	555.21	127.21	453.10
3	5.52	280.20	70.43	209.70	6.27	289.29	72.06	219.14
4	14.71	892.03	186.10	547.23	14.53	894.83	186.10	545.81
5	22.69	1125.64	256.99	949.48	24.07	1315.88	277.82	945.45
Average	13.49	596.01	159.56	542.05	14.28	735.82	169.36	541.85

## Conclusions

This paper focuses on the impact of demand surge on the distribution scheduling and route optimization of food cold chain. Food cold chain has the characteristics of small order volume, various types,nd high-temperature control requirements. Its distribution scheduling and route optimization problem have been extensively studied. In addition, in the event of online promotions and other activities, the number of orders has doubled in a short period of time. Considering the time requirements of cold chain products, we optimized the distribution scheduling to deliver products to customers in a short time with high efficiency and quality.

In order to solve the integrated optimization problem of food cold chain distribution scheduling and routing under demand surge, a mixed integer programming model with the shortest time and lowest cost as the objective function is established, and three-phase heuristics are designed to solve it. Through the actual data during the promotion of the enterprise, we compare the three-stage heuristic algorithm with the traditional algorithm; the delivery time and delivery cost have been significantly reduced, and the total delivery mileage has also been reduced by 5.8%, while the total load has not been reduced. The authenticity and effectiveness of the algorithm in this paper are verified. At the same time, it also gives a reference for the demand surge of enterprise.

Future research could be expanded from the direction of assumption and algorithm. First, this paper is for offline orders,where information of all orders is known, while research on online is more suitable for demand surge; whether there is a more efficient heuristic algorithm to solve this kind of problem is also worthy of further study. Also, the background of this paper is the promotion of e-commerce. There are still other situations where there is a small probability of demand surge, such as emergencies, and research on emergency events is also of great significance to reality.
